# The complete mitochondrial genome of *Ornithomya biloba* (Diptera, Hippoboscidae)

**DOI:** 10.1080/23802359.2022.2075286

**Published:** 2022-05-12

**Authors:** Xin Li, Liang Wang, Ding Yang

**Affiliations:** Department of Entomology, College of Plant Protection, China Agricultural University, Beijing, China

**Keywords:** Mitochondrion genome, *Ornithomya biloba*, Hippoboscoidea, phylogeny

## Abstract

The mitochondrial genome (mitogenome) of *Ornithomya biloba* (Dufour 1827) was first sequenced and annotated in this study as the first representative of the genus *Ornithomya*. The complete mitogenome is 18,654 bp in length and contains 37 genes (13 protein-coding genes (PCGs), 22 tRNA genes, two rRNA genes, and control region). The phylogenetic analysis based on 13 PCGs in IQ-TREE supports the monophyly of Hippoboscidae, which was a sister group of Streblidae. Families Hippoboscidae and Streblidae formed the monophyletic Hippoboscoidea clade.

The family Hippoboscidae is characterized by the obviously dorsoventrally flattened body shape, head sunk into the thorax, their parasitism, and blood-sucking feeding habit (Soós and Hůrka 1986; Xue and Chao 1996). Hippoboscidae is a specific parasite family that infects birds and mammals (bats, cows, sheep, etc.) (Soós and Hůrka 1986). Some hippoboscid adults are vectors that could spread diseases (Xue and Chao 1996). Due to parasitic activity, they can spread worldwide with their hosts. There were 67 genera and 775 accepted species in the world (https://www.catalogueoflife.org/; query date: 2021-06-02). Hippoboscid flies are parasitic to birds and mammals and thus play an important role in their ecosystems. Here, we sequenced and annotated the mitochondrial genome data of *Ornithomya biloba* (Dufour 1827) in Hippoboscidae and roughly explored their phylogenetic relationship with some related groups.

The specimens of *Ornithomya biloba* (voucher number: LX2018-16) were collected in Longtan Waterfall, Wuling Mountain, Hebei Province, China (117.466003 E, 40.60182 N) by Jinlong Ren on 2 June 2018, and identified by Xin Li. The specimens were deposited in the Entomological Museum of China Agricultural University, Beijing, China (Liang Wang, 1352659341@qq.com). The genomic DNA was extracted from the whole body (except wings) of the specimen using the DNeasy Blood & Tissue Kit (Qiagen, Hilden, Germany), and then the DNA sample was stored at a −20 °C refrigerator. The mitochondrial genome was sequenced on the Illumina NovaSeq 6000 platform by Novogene Co., Ltd. (Cambridge, UK). Quality control and assembling were conducted in MitoZ software. A Python script *circle_check.py* in MitoZ software Github repository (https://github.com/linzhi2013/MitoZ) was used to confirm mitochondrial genome completeness (Meng et al. 2019). Annotation was executed in MITOS2 webserver (Donath et al. 2019) and corrected by hand following Cameron (2014).

The complete mitochondrial genome (mitogenome) of *Ornithomya biloba* (GenBank accession number: MZ379837) is 18,654 bp, which contains 37 genes (13 protein-coding genes (PCGs), 22 tRNA genes, two rRNA genes, and control region). The gene structure of *O. biloba* is similar to previous dipteran mitogenome studies (Zhou et al. 2017; Li et al. 2019). The nucleotide composition of *O. biloba* is 41.8% of A, 37.4% of T, 7.6% of G, and 13.2% of C, and A + T content is 79.2%. Six PCGs were started with ATG codon; atp8 was started with ATC; nad2, nad3, nad5, and nad6 were started with ATT; cox1 using TCG as start codon, and nad1 was initialed by TTG codon. Eight PCGs were terminated with TAA stop codon, while nad3 and cytb genes were stopped at TAG codon and cox1, cox2, nad5 were ended at single T. All tRNA genes were predicted and folded as cloverleaf structures.

In our study, 13 PCGs of 18 species were used in phylogenetic analysis, the GenBank accession numbers are listed as follows: *Syrphus ribesii* MW091497 (Chen et al. 2021), *Melanostoma scalare* MT185683 (Liu et al. 2020), *Paralimna concors* MT938921 (Zhao et al. 2020), *Ilythea japonica* MT527723 (Wang et al. 2020), *Paradyschiria parvula* MK896865 (Trevisan et al. 2019), *Paratrichobius longicrus* MK896866 (Trevisan et al. 2019), *Ornithomya biloba* MZ379837 (present study), *Lipoptena grahami* MT679542 (Wang et al. 2021), *Melophagus ovinus* KX870852 (Liu et al. 2017), *Melophagus ovinus* MH024396 (Tang et al. 2018), *Gasterophilus intestinalis* KU236025 (Gao et al. 2016), *Gasterophilus pecorum* KU578262 (Zhang et al. 2016), *Elodia flavipalpis* JQ348961 (Zhao et al. 2013), *Ectophasia rotundiventris* MK644821 (Li et al. 2017), *Sarcophaga albiceps* KT444443 (Liao et al. 2016), *Sarcophaga impatiens* JN859549 (Nelson, Cameron, et al. 2012), *Calliphora vomitoria* KT444440 (Yan et al. 2014), *Calliphora vicina* JX913760 (Nelson, Lambkin, et al. 2012). All phylogenetic analyses were conducted in Phylosuite (Zhang et al. 2020), including sequences alignment in MAFFT (Katoh and Standley 2013), alignments trimming in trimAl (Capella-Gutierrez et al. 2009), substitution model selecting in ModelFinder (Kalyaanamoorthy et al. 2017), and maximum-likelihood phylogenetic tree rebuild in IQ-TREE (Minh et al. 2013; Nguyen et al. 2015). The topology and node support values are given in Figure 1. IQ-TREE analysis revealed all the outgroups diverged from the rest. Newly sequenced *Ornithomya biloba* was sister to *Lipoptena* and *Melophagus* genera clade. All Hippoboscidae was monophyletic as a sister group of Streblidae. Oestrioidea and Hippoboscoidea were assigned to be sister groups in our dataset.

**Figure 1.**
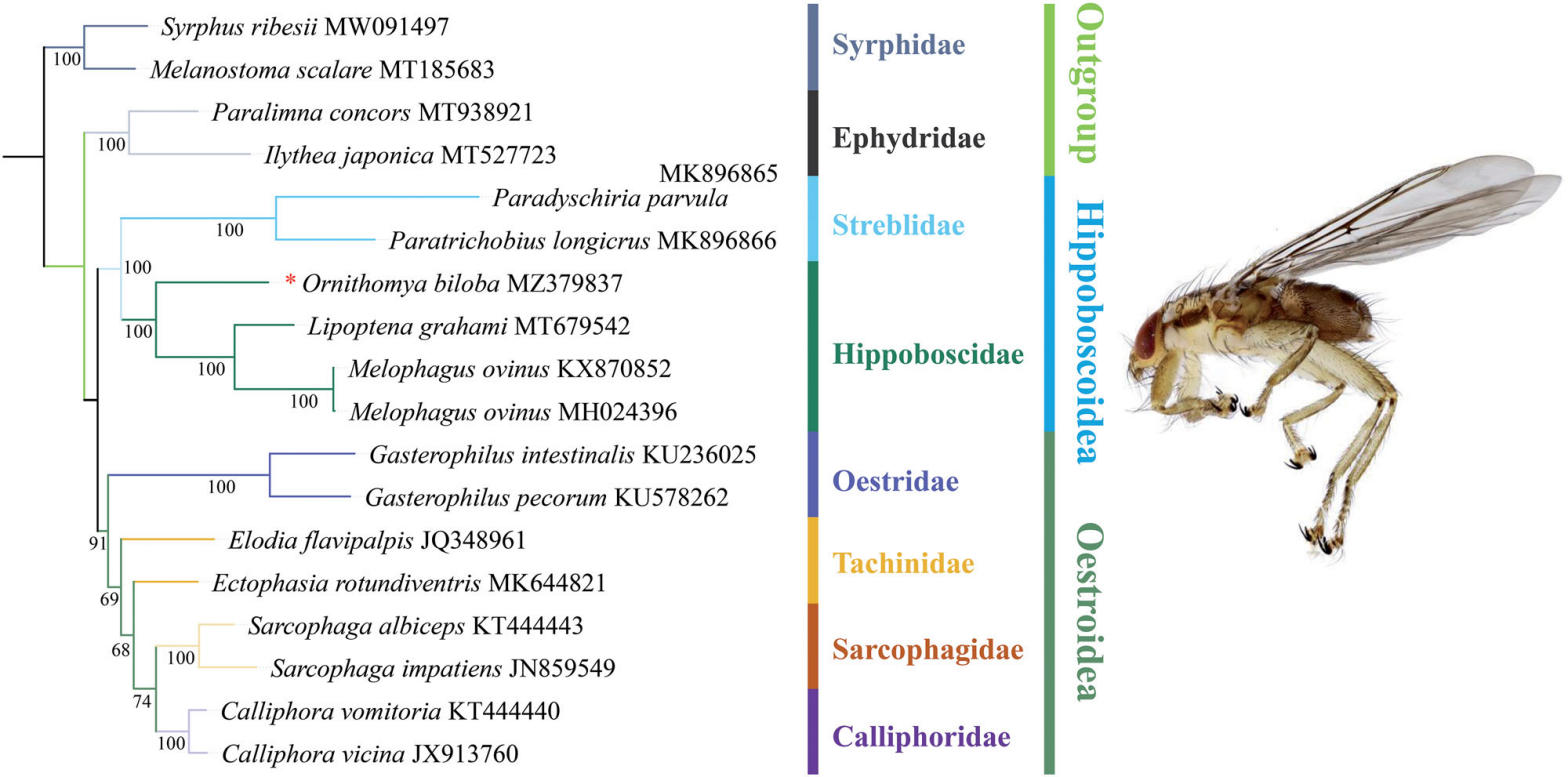
The maximum-likelihood phylogenetic tree of 17 fly species inferred by IQ-TREE based on 13 protein-coding genes. Asterisk indicates the data sequenced in this study.

## Data Availability

The mitochondrial genome used in this study is available in the GenBank of NCBI at https://www.ncbi.nlm.nih.gov/ under the accession number MZ379837. The associated BioProject, BioSample, and SRA numbers are PRJNA734480, SAMN19493608, and SRR14710877, respectively.
